# Additive Manufacturing of Polymer–Fiber Composites

**DOI:** 10.3390/ma15155337

**Published:** 2022-08-03

**Authors:** R. A. Ilyas, A. S. El-Shafay, M. T. Mastura, Shahir Mohd Yusuf, Emin Bayraktar, Abdul Hadi Azman

**Affiliations:** 1 School of Chemical and Energy Engineering, Faculty of Engineering, Universiti Teknologi Malaysia (UTM), Johor Bahru 81310, Malaysia; 2 Centre for Advanced Composite Materials, Universiti Teknologi Malaysia (UTM), Johor Bahru 81310, Malaysia; 3 Department of Mechanical Engineering, College of Engineering, Prince Sattam Bin Abdulaziz University, Al-Kharj 16273, Saudi Arabia; a.abdou@psau.edu.sa; 4 Mechanical Power Engineering Department, Faculty of Engineering, Mansoura University, Mansoura 35516, Egypt; 5 Faculty of Mechanical and Manufacturing Engineering Technology, Universiti Teknikal Malaysia Melaka, Hang Tuah Jaya, Durian Tunggal, Melaka 76100, Malaysia; 6 Engineering Materials and Structures (eMAST) iKohza, Malaysia-Japan International Institute of Technology (MJIIT), UTM Kuala Lumpur, Jalan Sultan Yahya Petra, Kuala Lumpur 54100, Malaysia; shahiryasin@utm.my; 7 ISAE-SUPMECA-PARIS, School of Mechanical and Manufacturing Engineering, 93400 Saint-Ouen, France; emin.bayraktar@isae-supmeca.fr; 8 Department of Mechanical and Manufacturing Engineering, Faculty of Engineering and Built Environment, Universiti Kebangsaan Malaysia, Bangi 43600, Malaysia; hadi.azman@ukm.edu.my; 9 Centre for Automotive Research, Faculty of Engineering & Built Environment, Universiti Kebangsaan Malaysia, Bangi 43600, Malaysia

*Additive Manufacturing of Polymer–Fiber Composites* is a newly open Special Issue of *Materials*, which aims to publish original and review papers on new scientific and applied research, and make great contributions to the finding and understanding of the fabrication of fiber-reinforced polymer composites using current advanced additive manufacturing techniques. This Special Issue also covers fundamentals, characterization, and applications of fiber-reinforced polymer composites.

The technology of the additive manufacturing (AM) process has significantly piqued the interest of researchers and industrial players from various areas [1,2,3,4,5]. Flexibility of this technology has increased the potential of research and exploration from the supply of materials until the end of life. Regarding the environmental aspect, the reduction in the need for raw materials in additive manufacturing will enhance the positive impact on the environment. Therefore, selection of the right eco-friendly sources of energy, recyclability and biodegradability, and maximizing the product’s end-of-life value is important in AM [6,7,8,9]. Fiber-reinforced polymer composites have been employed in the early development of AM technology, and they have further potential application in various types of product design [10,11]. Moreover, the research and development of these materials are extensively progressing as the materials are varied and have unique characteristics.

Studies on fiber-reinforced polymer composites for the additive manufacturing process have been carried out by many researchers and become an interesting topic to be explored in the additive manufacturing industry. The aims of the inclusion of fiber in the polymer-based feed stock materials are to reduce the percentage of polymers and consequently reduce the harmful substances that may emit during the fabrication process in AM. Moreover, fiber-reinforced polymer composites could improve the properties of the dominant materials and exhibit good performance in the final products. Employment of the fibers in the AM process should consider the type of fiber, fiber treatment, fiber size, fiber loading, and fiber characteristics. It is important to understand the composition of fibers to ensure their compatibility with the polymer, and how they could produce good resultant composites for AM.

The AM process starts from the preparation of the feed stock until the disposal of the fabricated parts [12]. The impact of this process towards the environment is a major concern of the industry since the AM process is known as an environmentally conscious manufacturing process, and consumption of the materials is optimum. However, due to the heating process of thermoplastic polymer that may cause adverse health effects towards humans, biopolymer and bio-composite materials are becoming alternative materials for the feedstock, and numerous studies had presented the advantages of these types of materials for AM. Process preparation of the biopolymers and bio-composites is less harmful and easy to dispose of at the end of its life. Hence, it is important to consider environmental elements during the AM process from the beginning until the end of life of the fabricated parts. Extensive studies should be conducted for various topic of additive manufacturing of fiber-reinforced polymer-based composites.

In this Special Issue, we aim to capture the cutting edge of the state-of-the-art research pertaining to advancing additive manufacturing of fiber-reinforced polymeric materials. The topic themes include advanced fiber-reinforced polymeric material development, processing parameter optimization, characterization techniques, structure–property relationships, process modelling, etc., specifically for AM.
